# Discovery of an Atypical Arp2/3 Complex in Malaria Parasites Sheds New Light on Nuclear Actin

**DOI:** 10.1002/cm.70030

**Published:** 2025-08-19

**Authors:** Franziska Hentzschel, Friedrich Frischknecht, Matthias Marti

**Affiliations:** ^1^ Integrative Parasitology, Centre for Infectious Diseases Heidelberg, University Medical Faculty Heidelberg Germany; ^2^ German Center for Infection Research DZIF, Partner Site Heidelberg Heidelberg Germany; ^3^ Institute of Infection and Immunity University of Glasgow Glasgow UK; ^4^ VetSuisse and Medical Faculties University of Zurich Zurich Switzerland

**Keywords:** actin, Arp2/3 complex, mitosis, *Plasmodium*

## Abstract

The Arp2/3 complex is a key actin nucleator essential for cytoskeletal dynamics in eukaryotes. Previously believed absent in apicomplexan parasites, we recently identified an atypical Arp2/3 complex in malaria parasites consisting of five divergent subunits and a putative kinetochore‐associated factor. This complex ensures proper kinetochore‐spindle attachment during male gametogenesis, likely by nucleating actin at the mitotic spindle. Disruption of Arp2/3 function or actin polymerization leads to defective DNA segregation into gametes and developmental arrest of the parasite in the mosquito. Our findings reveal unexpected diversity in Arp2/3 complex composition and function, highlighting specialized adaptations in malaria parasites and expanding our understanding of the Arp2/3 complex and actin functions during mitosis. Here, we discuss some of the open questions that need to be addressed to fully understand the molecular mechanism of this unusual Arp2/3 complex and its essential role in malaria transmission.

1

The actin‐related protein 2/3 (Arp2/3) complex is a central actin nucleator in most eukaryotic cells and the only one able to nucleate branched actin networks (Campellone et al. 2023; Gautreau et al. 2022). The complex consists of seven subunits: two actin‐related proteins Arp2 and Arp3, and five accessory proteins called ARPC1‐5. Nucleation of branched actin networks by the Arp2/3 complex is essential for many cellular processes, including cell morphology, cell motility, and endocytic trafficking (Campellone et al. 2023). Recently, nuclear functions of Arp2/3 have also been described, including DNA damage repair, nucleation of spindle actin from the centrosomes, and safeguarding chromosome segregation (Campellone et al. 2023; Hurst et al. 2019; Wollscheid and Ulrich 2023). The complex is assembled in an inactive state and activated by interaction with nucleation‐promoting factors (NPFs) belonging to the WASP family, which induce a conformational change enabling actin nucleation (Gautreau et al. 2022; Stradal et al. 2025). While the Arp2/3 complex usually binds to a mother filament to nucleate branching filaments, certain NPFs of the WISH/DIP/SPIN90 family can activate the Arp2/3 complex in the absence of a mother filament, resulting in the formation of linear actin filaments (Gautreau et al. 2022; Stradal et al. 2025).

The Arp2/3 complex is highly conserved across eukaryotes and present in animals, plants, and unicellular protists like the amoeba *Naegleria* and the ciliate *Tetrahymena* (Gordon and Sibley 2005; Kollmar et al. 2012; Velle and Fritz‐Laylin 2020) (Figure 1A). However, it was assumed that the complex is absent in the phylum Apicomplexa that includes many major human pathogens, given that no homologues to the canonical Arp2/3 subunits were identified in genome databases apart from a distant homologue to ARPC1 (Baum et al. 2006; Gordon and Sibley 2005). We set out to investigate the function and interaction partners of this remnant Arp2/3 complex member in the apicomplexan *Plasmodium berghei*, a rodent malaria model parasite. This led to the discovery of an atypical Arp2/3 complex with an unusual role in regulating kinetochore‐spindle attachment during mitosis (Hentzschel et al. 2025) (Figure 1B).

**FIGURE 1:**
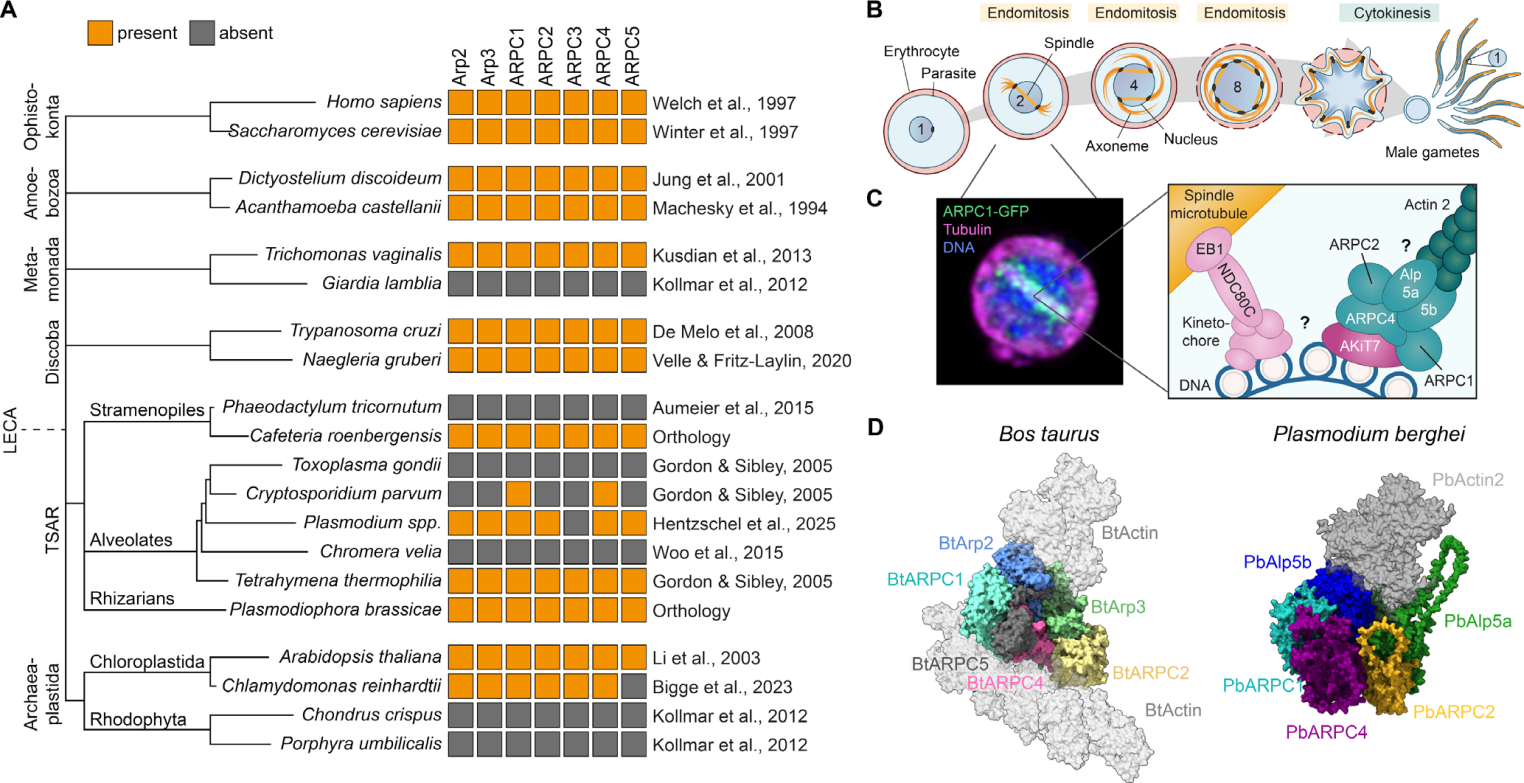
The atypical *Plasmodium* Arp2/3 complex. (A) Presence or absence of the Arp2/3 complex subunits in representative species across the eukaryotic tree of life based on published data and Uniprot annotation. Branch length of the phylogenetic tree is arbitrary. References for each species are given to the right. For *P. brassicae* and *C. roenbergensis*, Arp2/3 orthologues are annotated in the genome, but have not been investigated yet. Note that ARPC4 is present in *A. castellanii* according to experimental evidence, but the gene is not yet identified/annotated in the genome. LECA, last eukaryotic common ancestor. (B) Schematic of *Plasmodium* male gametogenesis. Numbers in nuclei indicate number of haploid genomes. Note that the entire process is completed in ~15 min. (C) ARPC1‐GFP localisation at the spindle of activated gametocytes (immunofluorescence microscopy, left) and model of Arp2/3 localisation at the kinetochore/spindle interphase (right). Question marks indicate unclear relationships and open research questions. Model adapted from (Hentzschel et al. 2025). (D) Structure comparison between the canonical Arp2/3 complex (bovine, left) and *Plasmodium* Arp2/3 (right). Bovine Arp2/3 structure from PDB accession ID 7TPT (Ding et al. 2022). *Plasmodium* Arp2/3 structure predicted with Alphafold 3 (Abramson et al. 2024). Note the overall positional similarity despite large extra loops and extensions present in *Plasmodium* Arp2/3 subunits.

*Plasmodium* parasites alternate between a vertebrate and a mosquito host. Right after uptake by a mosquito bite, male and female sexual precursor cells activate to form gametes. Male gametogenesis involves three rapid rounds of mitosis without nuclear division (Figure 1B). Only at the end are the eight genomes and nuclei separated as eight flagellated male gametes exit the residual body (Guttery et al. 2022; Rashpa and Brochet 2022). Generation of a parasite line expressing GFP‐tagged *Plasmodium* ARPC1 enabled us to localise the protein near the spindles during all three rounds of mitosis (Figure 1C). Genetic deletion revealed that ARPC1 was not necessary for parasite replication in the blood cells of the vertebrate host, but essential for development in the mosquito. Specifically, we found that ARPC1‐deficient parasites fail to correctly segregate their DNA during male gametogenesis, resulting in male gametes carrying on average only half a genome. These subhaploid gametes were still able to fertilise females and form functional zygotes, but parasites completely arrested in their development during later stages of the mosquito infection.

Co‐immunoprecipitation of GFP‐tagged ARPC1 identified only five interaction partners, two of which were the actin‐like proteins 5a and 5b (Alp5a, Alp5b), annotated as such based on their similarity to actin and difference to actin‐related proteins (Gordon and Sibley 2005). Using AlphaFold structural prediction, two of the three remaining interaction partners resembled the Arp2/3 subunits ARPC2 and ARPC4, respectively. Indeed, AlphaFold multimer predictions suggested that these five proteins (Alp5a/Arp3, Alp5b/Arp2, ARPC1, ARPC2 and ARPC4) arrange in the same orientation as their counterparts in the canonical Arp2/3 complex (Figure 1D). The third interaction partner was a protein annotated as Apicomplexan Kinetochore protein 7 (AKiT7), a protein previously found to be part of the unusually composed *Plasmodium* kinetochore, which links the spindle microtubules to the chromosomes (Brusini et al. 2022). The function of AKiT7, however, was not previously investigated. In the absence of ARPC1, kinetochores partially detached from the spindles and resided freely in the nucleus, suggesting that the uncoupling of chromosome linkage to spindle microtubules explains why only part of the genome was taken up into the emerging gametes during the final gamete formation.

Did we discover a *Plasmodium*‐specific Arp2/3 complex? To be a *bona fide* Arp2/3 complex, the identified complex would have to nucleate actin. To test this in the parasite, we expressed an F‐actin‐binding chromobody in *Plasmodium berghei* and found that it accumulated at the spindle during male mitosis and colocalised with ARPC1, indicating that actin filaments surround the spindle in a similar fashion as ARPC1. Also, inhibition of actin polymerisation using cytochalasin D phenocopied the ARPC1 knockout, resulting in kinetochore‐spindle detachment and formation of subhaploid male gametes. These results suggest that the atypical *Plasmodium* Arp2/3 complex indeed nucleates actin polymerisation in vivo, and that F‐actin is essential to ensure kinetochore‐spindle association and DNA segregation.

Multiple open questions remain after this discovery, both for *Plasmodium* biology and for Arp2/3 biology and evolution. Most notably, we identified only five subunits in *Plasmodium* Arp2/3 instead of the usual seven. It is unclear whether the missing two subunits, ARPC3 and ARPC5, are truly absent or just bind too loosely to the Arp2/3 complex to be pulled down during co‐immunoprecipitation. It is tempting to speculate that the extended N‐terminal domain of *Plasmodium* ARPC4 compensates for the lack of either of these two subunits. Given the large differences on a primary sequence level and the multiple insertions and loops in the predicted structures of *Plasmodium* Arp2/3 subunits, resolving the structure of the entire complex would likely inform on core structure–function relationships common to all Arp2/3 complexes as well as unique *Plasmodium*‐specific adaptations.

It is also unclear how the *Plasmodium* Arp2/3 complex is activated in the absence of recognizable *Plasmodium* homologues to WASP or WISH/DIP/SPIN90 family proteins (Kollmar et al. 2012). When we performed pulldowns on parasites that were not activated for gametogenesis, most complex members did not associate with each other, suggesting that the complex may only assemble upon activation of gametogenesis and might be immediately active. If confirmed, this would be a striking difference to canonical Arp2/3 that forms a stable, but inactive complex in the absence of NPFs (Gautreau et al. 2022). Whether yet unidentified proteins take the role of NPFs in *Plasmodium* (AKiT7 may be a candidate for this), or whether assembly and activation of the complex is linked to the phosphorylation signalling cascade known to coordinate induction of gametogenesis (Invergo et al. 2017) remains to be investigated. Notably, two serine positions within the Alp5a sequence were found to be phosphorylated within 60 s after activation of male gametogenesis (Invergo et al. 2017), providing a starting point for further investigations.

Finally, the molecular mechanism of how *Plasmodium* Arp2/3 and F‐actin safeguard kinetochore‐spindle attachment remains to be discovered. Notably, Arp2/3 seems only to be required for DNA segregation during male gametogenesis and not during the other replicative stages of the *Plasmodium* life cycle, indicating a specialised function. Male gametogenesis stands out for its extremely rapid mitosis: three rounds of DNA replication and mitosis happen within only 10 min within a single nucleus. Our time‐course immunofluorescence assays revealed that in the absence of ARPC1, all kinetochores initially attach to the spindle but progressively detach during the multiple rounds of mitosis. Is Arp2/3 nucleating a protective actin network surrounding the spindle that ensures that kinetochores stay attached during the rapid mitosis? Where would those actin filaments be anchored to, and are they branched or linear? *Plasmodium* parasites have two divergent isoforms of actin: actin 1 is ubiquitously expressed and takes on canonical roles in trafficking and motility; actin 2 is only expressed in mosquito stages, predominantly in male gametocytes, where it is essential for male gametogenesis (Douglas et al. 2024). While actin 1 forms only very short, linear actin filaments in vitro, actin 2 forms longer filaments (Lopez et al. 2023). Actin 2 has been shown to locate near the male mitotic spindle (Lopez et al. 2023) and might thus be the actin isoform nucleated by Arp2/3 during gametogenesis. Further research is needed to untangle the function of the two actin isoforms and their precise function at the spindle. Of note, deletion of a formin‐like protein called MISFIT in *Plasmodium* yielded the same phenotype as deleting ARPC1 (Bushell et al. 2009), suggesting an interplay between these two putative actin nucleators during mitosis.

In conclusion, the discovery of an atypical Arp2/3 complex in *Plasmodium* underlines the importance and diversity of this actin nucleator across the eukaryotic kingdom and raises the question if divergent Arp2/3 complexes exist also in other species previously thought to have lost this actin nucleator (Figure 1A). The role of *Plasmodium* Arp2/3 in safeguarding DNA segregation during mitosis fits well with recent data implicating Arp2/3 in chromosome segregation in metazoans (Burdyniuk et al. 2018; Haarer et al. 2023; Plessner et al. 2019), although the modes of action differ between species. Further investigation of structure–function relationships, the activation mechanism, and the molecular mechanism of action of this unconventional Arp2/3 complex will extend our knowledge about fundamental actin biology and may provide insights into the evolution and ancient roles of this complex.

## Author Contributions

Franziska Hentzschel: writing – original draft, writing – review and editing, visualisation. Friedrich Frischknecht: writing – review and editing. Matthias Marti: writing – review and editing.

## Conflicts of Interest

The authors declare no conflicts of interest.

## Data Availability

Data sharing not applicable to this article as no datasets were generated or analyzed during the current study.
